# Federated Semi-Supervised Multi-Task Learning to Detect COVID-19 and Lungs Segmentation Marking Using Chest Radiography Images and Raspberry Pi Devices: An Internet of Medical Things Application

**DOI:** 10.3390/s21155025

**Published:** 2021-07-24

**Authors:** Mahbub Ul Alam, Rahim Rahmani

**Affiliations:** Department of Computer and Systems Sciences, Stockholm University, 16407 Stockholm, Sweden; rahim@dsv.su.se

**Keywords:** internet of medical things, federated learning, semi-supervised machine learning, multi-task learning, transfer learning

## Abstract

Internet of Medical Things (IoMT) provides an excellent opportunity to investigate better automatic medical decision support tools with the effective integration of various medical equipment and associated data. This study explores two such medical decision-making tasks, namely COVID-19 detection and lung area segmentation detection, using chest radiography images. We also explore different cutting-edge machine learning techniques, such as federated learning, semi-supervised learning, transfer learning, and multi-task learning to explore the issue. To analyze the applicability of computationally less capable edge devices in the IoMT system, we report the results using Raspberry Pi devices as accuracy, precision, recall, $F_{score}$ for COVID-19 detection, and average dice score for lung segmentation detection tasks. We also publish the results obtained through server-centric simulation for comparison. The results show that Raspberry Pi-centric devices provide better performance in lung segmentation detection, and server-centric experiments provide better results in COVID-19 detection. We also discuss the IoMT application-centric settings, utilizing medical data and decision support systems, and posit that such a system could benefit all the stakeholders in the IoMT domain.

## 1. Introduction

With the ever-increasing availability of information, the connectivity among different electronic devices, and the transformation of the healthcare system, we have a new and promising research area: the internet of medical things (IoMT). IoMT refers to the effective interconnectivity among different medical devices to perform different healthcare operations [1,2,3]. With this goal, IoMT encompasses strategies, frameworks, applications, among other aspects [4]. The fundamental goal here is to improve the overall effectiveness, efficiency, and quality of different healthcare services and products [5].

Affordable healthcare for all, real-time monitoring and diagnosis, a patient-centric clinical approach, sustainable health and longevity, better doctor–patient interaction, and effective prevention of diseases are some of the critical applications of IoMT [6]. A simple but robust and secured structure, and cheap and user-friendly devices will ensure a significant increase in productivity [7], and be valuable for medical professionals to maintain the cases of many patients in a quick and organized manner [8,9]. It will also help the ever-increasing aging population (particularly in the west) and the decentralization of living areas from the city centers [10]. One of the critical aspects to ensure this success is the effective utilization of medical decision support systems or tools [11].

We have a plethora of medical data stored and transmitted from different medical devices now. The data are obtained from different medical facilities, as they keep the patient records in a digital format. The data are exceptionally heterogeneous, including patient demographic information, diagnoses, laboratory test results, medication prescriptions, clinical notes, and medical images [12,13]. With the help of machine learning, this wealth of information can be used to create different machine learning-based decision support systems, which can help healthcare professionals in various ways to make correct diagnostic decisions [14]. Machine learning is a collection of computational methods that can be used for future predictions, utilizing data for a specific task. When the parameters of a particular machine learning algorithm are estimated in a balanced way so that it can predict the outcome reasonably accurately, it is called “learning or training the model” [15]. The crucial issue we should consider here is security and privacy [16]. We must not allow the information to be breached.

Federated learning (FL) can help us in this regard. FL is a distributed machine learning approach where the data are stored in different devices, and the training scope of the data is limited to those devices. A central server is responsible for taking care of the model and updating it through the locally trained data sets. This approach ensures that data are always kept in the local devices, and the central server is never allowed to view it. Therefore, privacy is ensured. The effective aggregation of different locally trained models in the server is the key challenge here [17]. FL is a practical approach to ensure low operation cost, better ownership control, and minimal usage of data stored locally in different devices [18,19]. It is worth noting that there is a common assumption about FL, which is that the data stored in local devices are labeled with full annotations, so it can be used for machine learning easily with the supervised approach where all the data must be labeled. Unfortunately, this is often not the case, as labeling data is a costly endeavor, requiring medical professionals to do so [20].

Semi-supervised learning (SSL) and transfer learning (TL) could be used to tackle the data annotation scarcity issue. SSL can train the model, using both the labeled and unlabeled data at the same time [21]. TL can be used to transfer the knowledge from one trained model to another. It is also essential for domain knowledge transfer [22]. The combination of SSL and TL can be beneficial here, as it has been shown that both SSL and TL show impressive performance for computer vision and natural language processing tasks [23,24,25,26].

To utilize the techniques mentioned above in a practical setting for the IoMT application, we should consider the viability of training the models in the IoMT related low-end and high-end devices, emphasizing the low-end devices. This is because, in a general IoMT setting, it should be expected that most of the end devices are computationally less powerful, compared with the standard devices used in the traditional machine learning research areas. As we have highlighted in Section 2, most of the concerning works are aimed at supervised learning, and medical decision support cases are not investigated in detail in a semi-supervised manner. It can also be seen that the low-end device usage was not investigated adequately as well. Based on this discussion we can formulate the following research question for this study.

What is the comparative performance in joint multi-task based medical decision support related semi-supervised transfer learning between high-end and low-end devices in a federated learning setting?

To address this research question, we aim to perform two such medical diagnostic tasks using a multi-tasking learning approach to detect the practical aspects of using both high-end and low-end devices in terms of a performance evaluation, using different metrics, such as accuracy, precision, recall, $F_{score}$, and dice similarity score. We also report the temperature and memory usage for the low-end device usage to discuss the additional usability aspects of using low-end devices. The two medical tasks are detecting COVID-19 and lung segmentation using chest radiography images. COVID-19 is a new strain of the coronavirus that has created a pandemic all over the world. It can infect the lungs to cause deadly respiratory syndromes [27,28,29]. The detection of COVID-19 and lung segmentation are performed in a multi-task learning fashion, where we optimize both losses in a combined strategy to create a shared representation–learning for both tasks. For the low-end IoMT device, we used Raspberry Pi [30] devices to train the data in an FL approach. We also compared the results using a desktop server to report the case for the high-end devices. Our results indicate that although the overall performance is reasonable in terms of accuracy, the latency should be improved in the future.

## 2. Related Works

Kairouz et al. [17] identified heterogeneity of the devices (particularly the difference in hardware) as one of the platform development and deployment challenges, while discussing advances and open problems in federated learning. They also emphasized that device stability or performance should not be affected by the running computations. Li et al. [31] also identified the devices’ communication, storage, and computational capabilities in a federated learning-based setting as a core challenge. They, in particular, highlighted the hardware (CPU, memory) and power (battery level). Bonawitz et al. [32] highlighted the scaling issue of federated learning with the practical system design case and identified the scarce computational resources and relatively small storage capacity of the devices as a practical issue. Based on this discussion mentioned above, we can surmise that the challenges of utilizing low-end devices in a federated learning setting are well-identified in the recent research reviews.

Li et al. [31] highlighted the scarcity of labeled data in the practical federated learning setting. In order to tackle this issue, they emphasized investigating beyond the supervised learning cases as one of the potential future directions in this research area. Gao et al. [33] reported the performance in an FL setup using Raspberry Pi devices for speech commands and electrocardiogram-related tasks. Their main focus was on supervised learning. He et al. [34] integrated support for Raspberry Pi in their federated machine learning framework; however, they did not report any result for the device-centric case or the semi-supervised/transfer learning. Chen et al. [35] introduced a federated transfer learning framework for wearable healthcare and provided the use cases for human activity recognition and Parkinson’s disease detection tasks. The details about the computation machine were not mentioned there. Zhang et al. [20] attempted benchmarking of semi-supervised federated learning. Their primary focus was on benchmarking the general FL algorithms with respect to the common data sets, such as Cifar-10, SVHN, and EMNIST. The utilization of low-end devices was absent there. It is also noteworthy that several other semi-supervised attempts were taken, using medical image-related tasks [36,37,38,39]; however, the low-end device utilization was not a major concept there. Based on the discussion, it is evident that there is a noticeable gap related to the performance comparison between the high-end and low-end devices for medical diagnosis-related tasks in the federated semi-supervised and transfer learning-based settings.

## 3. Methods and Materials

In this section, we discuss the methods and data used to address the research question presented in Section 1 for the detection of COVID-19 and lung segmentation tasks.

### 3.1. Data

For the COVID-19 data, we used the COVID-19 Radiography Database [40,41]. This “COVID-19”, “normal”, and “other lung infection” data set was released in stages. The authors released 219 “COVID-19”, 1341 “normal”, and 1345 “viral pneumonia” chest X-ray (CXR) images in the first release. In the first update, they increased the “COVID-19” class to 1200 CXR images. In the 2nd update, they increased the database to 3616 “COVID-19” positive cases along with 10,192 “Normal”, 6012 “Lung Opacity” (non-COVID lung infection), and 1345 “Viral Pneumonia” images.

For the lungs-segmentation boundary detection task, we used the JSRT (Japanese Society of Radiological Technology) database [42]. It contains 154 nodule and 93 non-nodule chest radiography images with additional information, such as patient age, gender, diagnosis (malignant or benign), X and Y coordinates of nodule, simple diagram of nodule location, and degree of subtlety in the visual detection of nodules.

### 3.2. Federated Learning

**Figure 1:**
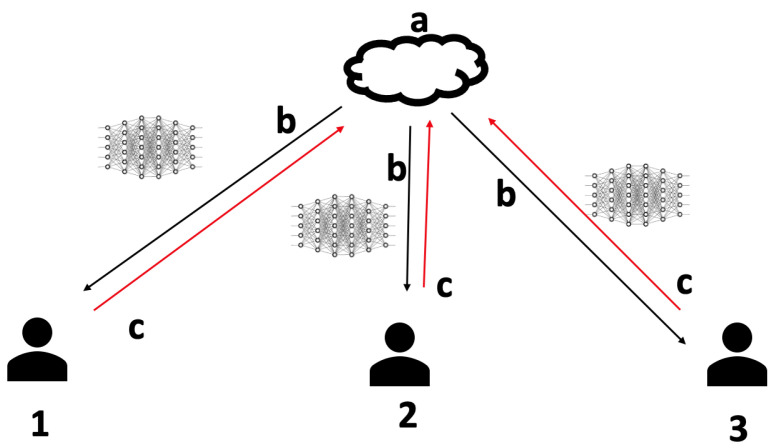
Federated learning architecture.

Figure 1 refers to the architecture that we used for the federated learning framework. Here, the global model is initialized by the server (a). The server then sends this model to all the client devices (b). After updating the model locally, the clients then send back the updated model to the server (c). The server then aggregates all the local models and updates the global model. One cycle from a–c is known as a round. The process is continued for several rounds. The following aggregation techniques are used.

#### 3.2.1. Simple Averaging (‘Simple’)

The global model is calculated as the simple average of all the local model weights in this method.

#### 3.2.2. Standard Deviation Based Weighted Averaging (‘std_dev’)

In this method, the local weights to be averaged based on the client’s validation metric (“accuracy” or “loss”) on their model with the validation data, and if the metric is greater than the difference between the average of evaluation metrics and standard deviation, then the weights are used for averaging, else the weights are discarded.

### 3.3. Semi-Supervised Multi-Task Learning

We used the architecture as described in [43] where a U-net [44] based encoder-decoder architecture was used. Encoder is defined using a convolutional neural network with pooling layers attached with fully-connected layers. Let segmentation images and classification images as $X^{s}$ and $X^{c}$ and their labels as *Y* and *C*. Let us assume the data distribution is unknown as $p(X^{s},Y)$ and $p(X^{c},C)$ for both cases. We sample (i.i.d.) labeled training sets from both of these distributions as $D_{l}^{s}$ and $D_{l}^{c}$. We also sample (i.i.d.) unlabeled training sets from $p(X^{s})$ and $p(X^{c})$ as $D_{u}^{s}$ and $D_{u}^{c}$. In this context i.i.d. refers to independently and identically distributed data, which indicates that the distribution from where we are drawing the data does not change.

Both the data augmentation and pseudo-labeling are used for the semi-supervised learning in unlabeled images. For the augmentation, both the strong and weak variants are used. Let $L_{l}$ be the cross-entropy based supervised loss, $\hat{c_{l}}$ is the prediction of input $x_{l}^{c}$, $c_{l}$ is the actual label, and $\hat{c_{s}}$ is the prediction from the strong augmentation, λ is the unsupervised classification loss weight, $L_{u}$ as unsupervised loss, the pseudo-labeling function is $argmax(\hat{c_{w}})\geq t)$, $\hat{c_{w}}$ is the predictions using weak augmentation, *t* is the threshold for pseudo-labeling. The classification loss then becomes the following:

(1) $$L^{c}=L_{l}\left(\hat{c_{l}},c_{l}\right)-\lambda L_{u}\left(\hat{c_{s}},argmax\left(\hat{c_{w}}\right)\geq t\right)$$

Obtaining the gradients from the encoders, the saliency maps of the predicted classes were generated. During the decoding time these maps were used to dictate the segmentation. The input images are adjoined with the maps, then downsampled, and finally connected with the feature maps. KL divergence is also calculated in between labeled and unlabeled segmentation predictions to ensure consistency. Let segmentation loss weight be α, calculated from the dice loss; labeled segmentation image predictions are $\hat{y_{l}}$, actual corresponding labels are $y_{l}$, unsupervised segmentation loss weight is β, and unlabeled segmentation predictions are $\hat{y_{u}}$. The segmentation loss then becomes the following:

(2) $$L_{s}=\alpha L_{l}\left(\hat{y_{l}},y_{l}\right)+\beta L_{u}\left(\hat{y_{l}},y_{u}\right)$$

## 4. Experimental Setup

For the classification data set, we selected 7632 chest radiography images with equal positive and negative distribution for COVID-19. We then split the data into train, test, and validation sets with 80%, 10%, and 10% ratios. In another data set, the train data were split into two sets for transfer learning. For the initial training, we used 3204 images, and for transfer learning using a federated learning approach, we used 2000 images with equal positive and negative distribution for COVID-19. The segmentation data set contains 246 chest radiography images. We split it into train and test sets with 90% and 10% ratios. We used 100 training images for the transfer-learning-segmentation data set, and the rest training images were used for the initial training for the second data set.

The number of labeled and unlabeled images for each task-related dataset was selected as a 50% ratio for each. The initial learning rate was selected as 0.0001. Adam optimizer having adaptive learning rates of 1.0 was applied in every eight epochs. LReLU was used with a negative slope of value 0.2. We used 0.25 as the dropout value. The values for *t*,λ, α, and β were selected as 0.7, 0.25, 5.0 and 0.01.

**Figure 2:**
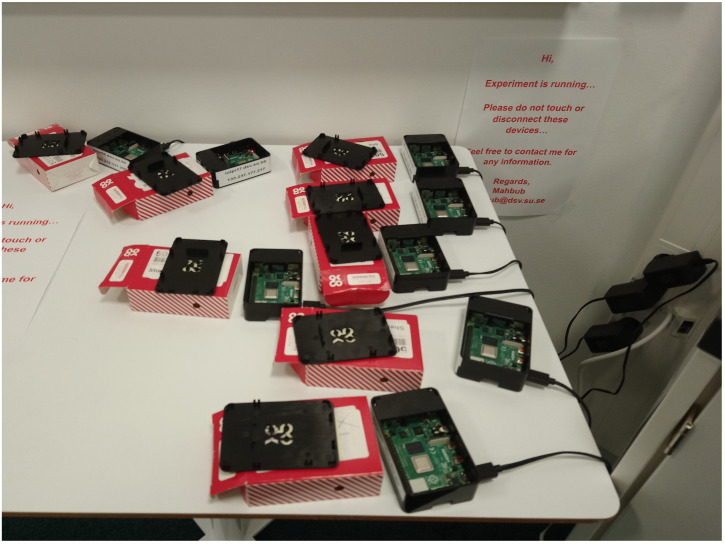
Eight Raspberry Pi devices were used to conduct the experiments.

In order to investigate the difference between the performance aspects and results between low-end edge devices, such as Raspberry Pi, and high-end computing devices, such as GPU-enabled servers, we tried to run the same experiments on both devices. A Ubuntu 18.04.5 LTS (GNU/Linux 4.15.0-142-generic x86_64) based server with GeForce RTX 2080 Ti GPU was used for the server-centric experiments. We used eight different Raspberry Pi 4 devices (Ubuntu 20.10 as GNU/Linux 5.8.0-1024-raspi aarch64) for the federated learning training (as shown in Figure 2). Selecting this number is an empirical one, as we had eight devices during the experimentation period. We could not employ a combination analysis of different devices due to time constraint issues regarding the training period (as discussed in Section 6). Python with PyTorch framework was used.

### 4.1. Experiments

We limit the rounds for the server-centric experiments to five and ten. For the Raspberry Pi-centric experiments, it is fixed as ten. Choosing these numbers is highly empirical, as they provided acceptable results after finishing the training. It could be argued that different combinations of these rounds could provide better results; however, as our primary concern in this study is to observe the performance difference between high-end and low-end devices, therefore we kept limited ourselves with these fixed values. Ideally, one should not test the trained model unless all the rounds are completed in training, as that could “expose” the model to the test data. Therefore, we cannot do a round-wise test in that manner. However, to ensure that the model is not overfitting or underfitting, the validation results obtained using the validation data set with the validation metrics (“accuracy” or “loss”) are always compared with the results obtained through the training data set in each epoch in every round. If the result comparison is too similar or different, then the previous best validation model is discarded.

For the server-centric experiments, we used the minibatch size as ten for both task-related datasets. We used the minibatch size of five for the Raspberry Pi-centric experiments for the classification images and one for the segmentation images. The simple averaging and standard deviation-based weighted averaging aggregation methods were used along with the evaluation metrics accuracy and loss. In order to investigate the performance difference between semi-supervised federated learning and semi-supervised federated transfer learning, we have conducted two experiments. They are discussed as follows.

#### 4.1.1. Semi-Supervised Federated Learning

This experiment uses all the training data distributed among different client devices to perform semi-supervised federated learning. We also simulate the client environments in the server.

#### 4.1.2. Semi-Supervised Federated Learning with Transfer Learning

In this experiment, we train the model with a portion of the training data in a single server in a semi-supervised manner. Then, with that trained model, we apply the federated learning approach with the rest of the training data equally distributed in different client devices to improve the performance.

### 4.2. Evaluation Metrics

For the COVID-19 classification task, we report the accuracy, precision, recall, and $F_{score}$. Accuracy is the ratio of the number of correct predictions to the total number of input samples. It works well only if there are an equal number of samples belonging to each class. For example, there are 98% samples of class *A* and 2% samples of class *B* in our training set. Then our model can quickly obtain 98% training accuracy by simply predicting every training sample belonging to class *A*. When the same model is tested on a test set with 60% samples of class *A* and 40% samples of class B, then the test accuracy drops down to 60%. The real problem arises when the cost of misclassification of the minor class samples is very high. If we deal with a rare but fatal disease, the cost of failing to diagnose the disease of a sick person is much higher than the cost of sending a healthy person to undergo more tests.

(3) $$Accuracy=\frac{Number\;of\;correct\;predictions}{Total\;number\;of\;predictions\;made}$$

If we have the predicted results, then the precision is the fraction of predictions that are relevant and correct, recall is the fraction of all relevant values that are predicted, and $F_{score}$ is the harmonic mean of precision and recall. These values of these measures can be calculated using true positives (TP), true negatives (TN), false positives (FP) and false negatives (FN), each representing one of the possible outcomes for binary classification, with a positive and a negative class. A true positive is when the machine learning model assigns the positive class to a positive example, and a false positive is when the model assigns the positive class to a negative example. Similarly, a true negative denotes the negative class predicted for a negative example, and a false negative when the negative class is selected for a positive example.

(4) $$Precision=\frac{TP}{TP+FP}$$

(5) $$Recall=\frac{TP}{TP+FN}$$

(6) $$F_{score}=\frac{2*Precision*Recall}{Precision+Recall}$$

As we are reporting four different evaluation metrics, including accuracy, precision, recall, and $F_{score}$ for the COVID-19 classification task, we consider accuracy as the primary evaluation metric for this task. This is because we are using equal distribution for positive and negative cases for training, testing, and validating the data. The other metrics are presented to indicate the performance robustness as if they are deviating much compared to accuracy; then, the performance may not be balanced. To elaborate on this, we need to consider that the medical data are often not equally distributed (much more positive data than negative data), and there is a different interest in terms of the prediction of the diagnosis. If we are mostly interested in the true positive cases and if the cost of the false positive is very high, then precision should be the ideal evaluation metric for the prediction task. If we do not want to reward false negatives, then recall should be the ideal choice. Usually, a balance between these two metrics is better for the overall evaluation. In this aspect, $F_{score}$ could be a helpful metric.

For the evaluation of the lung area segmentation detection task we report the dice similarity score. It can be calculated as between two images, as 2* the Area of Overlap divided by the total number of pixels in both images.

We report the temperature and CPU usage of all the Raspberry Pi devices in an average manner. We obtained the temperature value using Python’s CPUTemperature() function from the CPUTemperature library. The Linux ’free-h’ command was used to compute the active memory usage during the training time.

## 5. Results

Table 1 shows the results obtained from the server-centric federated semi-supervised learning experiments. Here, five clients were used. In total, five rounds of training were performed. In each round, there were five epochs of training. We can see that aggregation technique ’std_dev’ with validation evaluation of “loss” provides the best results.

Table 2 shows the results obtained from the initial model trained in the server, which will be used later to improve the performance on a transfer learning basis. Both best results using 10 and 15 epochs are obtained using the evaluation metric “loss”.

Table 3 shows the results obtained from the server-centric federated semi-supervised transfer learning experiments with an initial model obtained through training 10 epochs. Results show that with 10 rounds of federated learning based training, the overall result are improved.

Table 4 shows the results obtained from the server-centric federated semi-supervised transfer learning experiments with an initial model obtained through training 15 epochs. The results show that with 10 rounds of federated learning based training, the overall result is improved.

Table 5 shows the results obtained from the Raspberry Pi-centric federated semi-supervised learning experiments. We can see that standard deviation-based averaging aggregation technique provides slightly better performance over the “simple” averaging technique.

Table 6 shows the results obtained from the Raspberry Pi-centric federated semi-supervised transfer learning experiments. We can see that initial model trained with 15 epochs provides better performance.

Table 7 shows all the test results after each round for the Raspberry Pi trained models where the evaluation metric is “loss”, and the aggregation technique is “simple”, averaging where the transfer learning was used.

Table 8 shows all the test results after each round for the Raspberry Pi trained models where the evaluation metric is “loss”, and the aggregation technique is “simple”, averaging where only semi-supervised learning was used.

**Figure 3:**
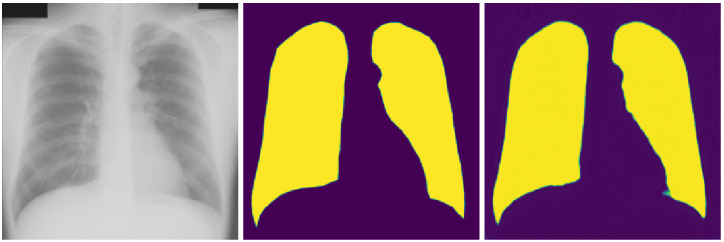
Best segmentation dice score (0.941) visualization, (**left**): original image, (**middle**): ground truth, (**right**): predicted segmentation.

**Figure 4:**
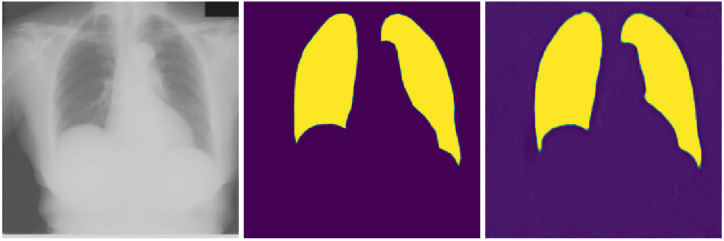
Worst segmentation dice score (0.776) visualization, (**left**): original image, (**middle**): ground truth, (**right**): predicted segmentation.

Figure 3 and Figure 4 show the segmentation task-related results from the Raspberry Pi-centric federated semi-supervised transfer learning experiment with initial training epoch ten, and the aggregation technique is “simple” averaging. The average dice score for this case is 0.884, as we can see in Table 6. The best dice score obtained here is 0.941, and the worst score is 0.776.

**Figure 5:**
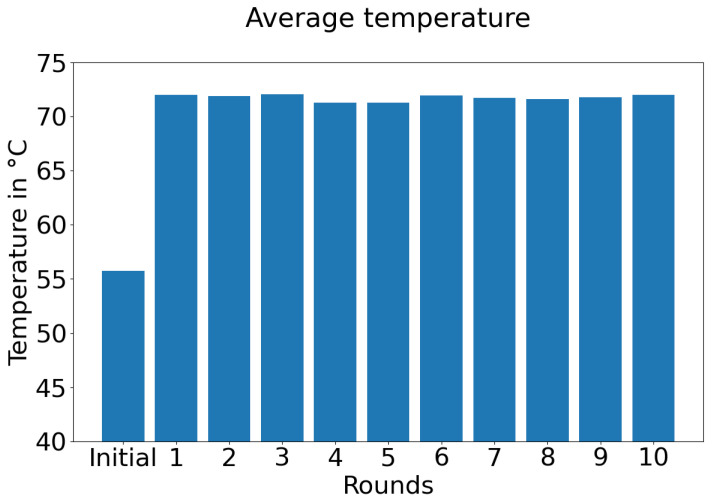
Average temperature in Raspberry Pi devices from federated semi-supervised learning experiment with aggregation technique “simple”.

Figure 5 displays the average temperature in Raspberry Pi devices from federated semi-supervised learning experiment with aggregation technique “simple”.

**Figure 6:**
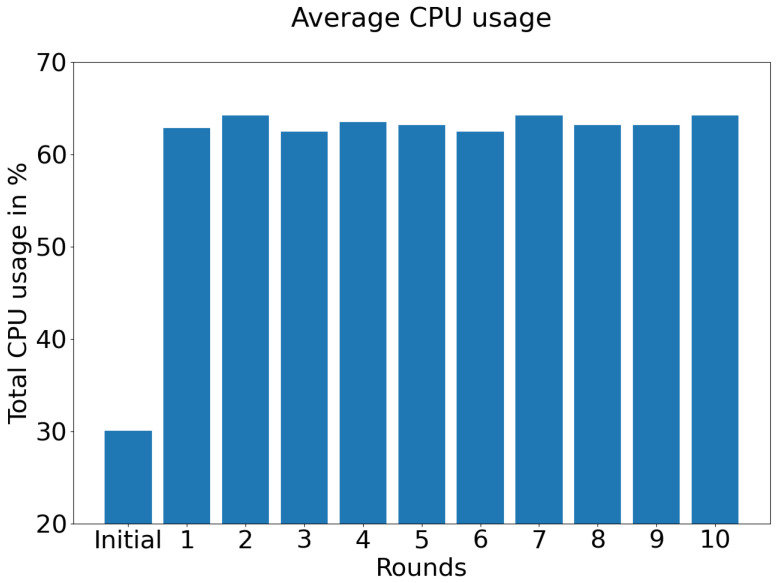
Average CPU usage in Raspberry Pi devices from federated semi-supervised learning experiment with aggregation technique “simple”.

Figure 6 displays the average CPU usage in Raspberry Pi devices from federated semi-supervised learning experiment with aggregation technique “simple”.

## 6. Discussion

We can see the performance comparison of semi-supervised federated learning between high-end and low-end devices in Table 1 and Table 5. Accuracy is comparatively lower in Raspberry Pi-centric results for the COVID-19 detection task, and the average dice score is similar in the chest boundary segmentation task. Given the computational difference in both device types, the result should be regarded as satisfactory, as relatively more straightforward aggregation methods were used here. If we use a relatively better model as the initial model for transfer learning, it should indicate superior performance. Comparison of the results presented in Table 3 and Table 4 validates this hypothesis in terms of accuracy. We can notice that the average dice score is decreased in Table 4. As the segmentation training data were relatively smaller, this could be an indication of slight overfitting. Comparison between Table 4 and Table 6 provides us the performance difference in federated semi-supervised transfer learning in high-end and low-end devices. Here, the accuracy is significantly low in Raspberry Pi-centric learning, compared to server-centric learning. The average dice score is better in Raspberry Pi-centric learning. The better usage of transfer learning techniques could improve the result here, which we will investigate in the future. The less number of mini-batch sizes in the Raspberry Pi-centric training could be another reason for the relatively poor performance compared with server-centric experiments. Therefore, we will explore this converging issue in the future. It can be observed that using the evaluation-metric as “loss” with the aggregation technique as “std_dev” provides superior results. In the future, we will explore further this aggregation optimization and better the hyper-parameter tuning issue. Therefore, the traditional training, validation, and testing paradigm should also be explored, as it is an FL approach.

Two critical issues can be observed by analyzing the performance of the different models mentioned above. GPU-vs-CPU trade-off is one key issue here in terms of performance evaluation. GPUs are notably faster due to the bandwidth issue, as it takes a considerably larger amount of memory for the CPU to train a model, due to its sequential job scheduling and fewer cores than GPUs. It is also essential to consider that optimizing could be more straightforward in CPU due to its architecture. In the case of irregular computations with smaller batch sizes, GPU performs better; on the other hand, for significantly large batch size, CPU could be better [45]. Therefore, if the performance is relatively similar, then the result obtained from the CPU should be acceptable if we exclude the longer training time constraint. Another issue that we have observed is the relatively poor performance when applying transfer learning. We argue that using a relatively smaller dataset for the initial model training and the absolute domain similarity with the destination task (and data)could be the reasons. The initial model training data should be considerably bigger in order to get better performance. The similarity in domains for both the tasks tends to cause overfitting, which also could be an issue here [46]. In the future, we will be careful about these issues.

As discussed in Section 4.1, so far, we have evaluated the performance only after all the rounds are completed. As the final round-number selection is a practical choice, the following question may arise: what if we have already achieved better performance in one of the previous rounds? To investigate this issue, we report the round-centric results in Raspberry Pi devices as shown in Table 7 and Table 8. In Table 7, we can see that we have already achieved better accuracy after round six in the case of transfer learning. In the case of semi-supervised learning, however, the last round provides the best accuracy (Table 8). In both cases, the average dice score progressed smoothly from the starting round to the finishing round. This indicates that there is a trade-off in selecting suitable rounds and relative performance in a multi-task learning setting.

We have two tasks in our joint multi-task-based learning setting: COVID-19 detection and lungs segmentation marking. Figure 3 and Figure 4 provide the best and worst result example in terms of dice score. Given the minimal data we had for the segmentation marking, the result is satisfactory. The relatively smaller amount of data also raises the possibility of overfitting in training. In the future, we will be careful about this issue.

As we are mostly interested in the performance of low-end devices in terms of their usability, we also investigated three additional aspects: training time, temperature, and CPU usage. We faced the primary issue while training using Raspberry Pi devices of the total time it takes to finish the training. On average, it took three hours to finish one round. In comparison, it took only thirty minutes to complete ten rounds in server-centric training. It is one of the key reasons we could not explore more in terms of hyper-parameter tuning. In the future, we will explore more on the effective and fast utilization of different edge-centric devices and techniques, as at present, we are limiting ourselves with TCP-IP connection as the socket only. Figure 5 indicates that the temperature becomes significantly high during the experiment time. It should be taken care of, as long-term high temperatures may damage the device or make the training process even slower. We can see that even with a minimal mini-batch size, the CPU usage is quite significant, as shown in Figure 6. It indicates that increasing the mini-batch size may hamper the overall process; therefore, we need to find superior techniques which should be used in a better CPU usage manner.

Hospitals regularly accumulate enormous amounts of patient data using various isolated medical equipment. These data comprise diagnosis results, images, unstructured text, and vital signs. Sometimes it is not possible to combine and store all of the data effectively so that it can be used or analyzed later. Interconnection of different medical equipment over the internet with the effective formation of a distributed platform such as IoMT can be a solution. Combining and utilizing [47] medical data from different sources can provide superior diagnosis and distinguish effective action for the patient in a fast and more effective way. Moreover, it provides the opportunity to create broader scale health networks among different hospitals or countries to improve patients’ health globally and platform. However, medical data is susceptible, and therefore the significant concerns in IoMT need to be addressed, such as reliability, safety, and security [48]. Therefore, it is evident that to tackle this issue we need to focus on the effective construction of the application domain of IoMT. The components of this application domain can be addressed as advanced level machine learning and deep learning [49], reasoning [50], natural language processing [51], speech recognition [52] and computer vision (image object recognition) [53], human-computer interaction, and dialog and narrative generation. From a global perspective, this can be used to incorporate the new generation hardware and software systems that imitate the human brain and cognitive functionality and thus enhance the human decision-making process. Therefore, we can view the IoMT application domain as a novel computational approach to create a more robust and accurate model resembling how the human brain senses, reasons, and response to various stimuli. IoMT applications should combine data analysis and multi-agent-based adaptive user interface (AUI) [54] to tailor the task and platform for the intended audience (the various stakeholders in the healthcare sectors in this case). By doing this, we hope that IoMT can overcome the issues of reliability, safety, and security by providing a system that is adaptive, interactive, iterative, stateful, and contextual.

In order to show the applicability of such an IoMT application, in this paper, we used chest-radiography data to identify COVID-19 and lung-segmentation, using the same semi-supervised machine learning architecture. We also aimed to show that the FL approach could be a valuable resource to deploy such architecture on a real-time basis using low-end devices such as Raspberry Pi. Although our results indicate a clear superiority in using high-end computational devices, if we consider the long-time deployment, privacy and security, cost-benefit analysis, and the distributed aspect, it is worthwhile to use such low-end devices for these IoMT applications.

Data intelligence is a critical aspect that we have to explore more in the future, as efficient usability largely depends on the quality of the data [55]. Data can be a wealth of resources if they can be adequately represented to tackle a healthcare problem such as urinary tract infection detection [56]. The effective surveillance deployment [57] also depends on the more nuanced representation of it. As IoMT deals with the effective integration of the medical equipment and its associated data, a careful exploratory analysis of this heterogeneous data and their applicability should be investigated together. Concise and practical IoMT data aggregation framework [58] should be analyzed as well. Many medical conditions or diseases are interrelated (co-morbidity), and the diagnosis process is relatively sparse with different medical equipment and data. Moreover, it is very crucial to consider the amount of time spent in some cases as well. For instance, sepsis detection [59,60] could be one such issue. It has also been argued that analyzing only one part of the whole data gathered for a diagnosis is not convincing enough to predict a particular disease, therefore using only chest radiography images to detect COVID-19 has also been criticized as it may not be quite convincing from a medical explanation perspective [61]. As we have discussed in Section 2 that federated semi-supervised learning in low-end devices for the medical decision support systems is not quite highlighted; therefore, we opted out of benchmarking comparison of this work. In the future, we will perform these benchmarking tasks along with the insights and improvements suggested from this study, subsequent observations, and discussion. Considering these aspects, we hope that a multi-modality-based [47] superior data representation combined with real-time data provided by the sensor [62] and superior edge device [63] integrated IoMT system with a user-friendly and patient-centric healthcare application and the existing open-source medical data could provide a better diagnostic helping tool for healthcare professionals and patients.

## 7. Conclusions

We investigated COVID-19 detection and lung segmentation detection problems from chest radiography images using various recent machine learning strategies, such as federated learning, semi-supervised learning, transfer learning, and multi-task learning. We compared the IoMT setup deployed using Raspberry Pi devices with high-end computational device-based experiments and found that, although for lung segmentation detection Raspberry Pi provides better results, it is slightly worse for COVID-19 detection. We also explored additional performance aspects, and we posit that a more nuanced representation of data with the practical construction of a superior IoMT framework would provide a better automatic diagnostic aiding tool for the stakeholders.

## Figures and Tables

**Table 1:** Results of server-centric federated semi-supervised learning experiments. The best performance is highlighted in bold.

Validation Metric	Aggregation Technique	Accuracy	Precision	Recall	$F_{\mathrm{score}}$	Average Dice Score
**loss**	**std_dev**	**0.720**	**0.666**	**0.883**	**0.759**	**0.844**
loss	simple	0.680	0.637	0.833	0.722	0.769
accuracy	std_dev	0.655	0.677	0.591	0.631	0.708
accuracy	simple	0.717	0.682	0.814	0.742	0.795

**Table 2:** Results of server-centric initial model training for transfer learning experiments. The best performance is highlighted in bold.

Total Epochs	Validation Metric	Accuracy	Precision	Recall	$F_{\mathrm{score}}$	Average Dice Score
10	accuracy	0.647	0.780	0.410	0.538	0.767
**10**	**loss**	**0.782**	**0.787**	**0.773**	**0.780**	**0.798**
**15**	**loss**	**0.802**	**0.810**	**0.790**	**0.800**	**0.780**
15	accuracy	0.761	0.817	0.673	0.738	0.708
5	loss	0.697	0.804	0.521	0.632	0.785
5	accuracy	0.625	0.618	0.655	0.636	0.737

**Table 3:** Results of server-centric federated semi-supervised transfer learning experiments with an initial model obtained through training 10 epochs. The best performance is highlighted in bold.

Total Rounds	Validation Metric	Aggregation Technique	Accuracy	Precision	Recall	$F_{\mathrm{score}}$	Average Dice Score
5	loss	simple	0.783	0.728	0.905	0.807	0.834
5	accuracy	std_dev	0.778	0.761	0.812	0.786	0.828
5	accuracy	simple	0.762	0.787	0.718	0.751	0.827
5	loss	std_dev	0.740	0.667	0.959	0.787	0.842
10	loss	simple	0.796	0.734	0.928	0.820	0.867
**10**	**loss**	**std_dev**	**0.814**	**0.792**	**0.852**	**0.821**	**0.867**
10	accuracy	simple	0.758	0.867	0.610	0.716	0.823
10	accuracy	std_dev	0.771	0.877	0.630	0.733	0.837

**Table 4:** Results of server-centric federated semi-supervised transfer learning experiments with an initial model obtained through training 15 epochs. The best performance is highlighted in bold.

Total Rounds	Validation Metric	Aggregation Technique	Accuracy	Precision	Recall	$F_{\mathrm{score}}$	Average Dice Score
5	loss	simple	0.772	0.744	0.829	0.784	0.806
5	accuracy	std_dev	0.809	0.770	0.881	0.822	0.807
5	accuracy	simple	0.730	0.664	0.931	0.775	0.812
5	loss	std_dev	0.749	0.688	0.912	0.784	0.815
10	loss	simple	0.820	0.830	0.804	0.817	0.835
**10**	**loss**	**std_dev**	**0.827**	**0.788**	**0.895**	**0.838**	**0.828**
10	accuracy	simple	0.759	0.867	0.612	0.717	0.823
10	accuracy	std_dev	0.769	0.875	0.628	0.732	0.837

**Table 5:** Results of Raspberry Pi-centric federated semi-supervised learning experiments. The best performance is highlighted in bold.

Aggregation Technique	Accuracy	Precision	Recall	$F_{\mathrm{score}}$	Average Dice Score
**simple**	**0.694**	**0.663**	**0.789**	**0.721**	**0.844**
std_dev	0.683	0.633	0.872	0.733	0.785

**Table 6:** Results of Raspberry Pi-centric federated semi-supervised transfer learning experiments. The best performance is highlighted in bold.

Initial Training Epochs	Aggregation Technique	Accuracy	Precision	Recall	$F_{\mathrm{score}}$	Average Dice Score
10	simple	0.725	0.656	0.949	0.775	0.884
**15**	**simple**	**0.760**	**0.697**	**0.920**	**0.793**	**0.855**
10	std_dev	0.723	0.651	0.964	0.777	0.883
15	std_dev	0.761	0.699	0.917	0.793	0.854

**Table 7:** Round-wise test results obtained from Raspberry Pi-centric transfer learning-based training. The best performance is highlighted in bold.

Total Rounds	Accuracy	Precision	Recall	$F_{\mathrm{score}}$	Average Dice Score
1	0.718	0.661	0.898	0.761	0.813
2	0.704	0.641	0.927	0.758	0.823
3	0.715	0.649	0.936	0.767	0.833
4	0.720	0.657	0.920	0.767	0.842
5	0.717	0.653	0.924	0.765	0.850
**6**	**0.725**	**0.661**	**0.923**	**0.770**	**0.858**
7	0.722	0.654	0.941	0.772	0.865
8	0.716	0.651	0.931	0.766	0.871
9	0.714	0.647	0.945	0.768	0.878
**10**	**0.725**	**0.656**	**0.949**	**0.775**	**0.884**

**Table 8:** Round-wise test results obtained from Raspberry Pi-centric federated semi-supervised learning-based training. The best performance is highlighted in bold.

Total Rounds	Accuracy	Precision	Recall	$F_{\mathrm{score}}$	Average Dice Score
1	0.533	0.522	0.801	0.632	0.701
2	0.552	0.549	0.584	0.566	0.733
3	0.581	0.574	0.628	0.600	0.753
4	0.593	0.586	0.638	0.611	0.770
5	0.624	0.610	0.691	0.648	0.786
6	0.647	0.630	0.713	0.669	0.800
7	0.661	0.641	0.731	0.683	0.813
8	0.678	0.654	0.756	0.701	0.825
9	0.692	0.654	0.815	0.726	0.835
**10**	**0.694**	**0.663**	**0.789**	**0.721**	**0.844**

## Data Availability

COVID-19 Radiography Database [40,41]: Official webpage link (https://www.kaggle.com/tawsifurrahman/covid19-radiography-database). JSRT (Japanese Society of Radiological Technology) database [42]: Official webpage link. (http://db.jsrt.or.jp/eng.php).

## Abbreviations

The following abbreviations are used in this manuscript:

IoMT	Internet of Medical Things
FL	Federated Learning
SSL	Semi-supervised Learning
TL	Transfer Learning
COVID-19	Coronavirus Disease 2019
	[Official name for the disease caused by the SARS-CoV-2 (2019-nCoV) coronavirus]
JSRT	Japanese Society of Radiological Technology
LTS	Long Term Support
GPU	Graphics Processing Unit
CPU	Central Processing Unit
AUI	Adaptive User Interface
